# Tracing potential soil contamination in the historical Solvay soda ash plant area, Jaworzno, Southern Poland

**DOI:** 10.1007/s10661-015-4930-7

**Published:** 2015-10-26

**Authors:** Katarzyna Sutkowska, Leslaw Teper, Monika Stania

**Affiliations:** Department of Applied Geology, Faculty of Earth Sciences, University of Silesia, ul. Bedzinska 60, 41-200 Sosnowiec, Poland

**Keywords:** Soda ash, Waste, Heavy metals, Soil contamination

## Abstract

This study of soil conditions was carried out on 30 meadow soil (podzol) samples from the vicinity of the soda ash heap in Jaworzno, supplemented by analyses of 18 samples of waste deposited on the heap. In all samples, the total content of macroelements (Ca and Na) and heavy metals (Cd, Cr, Ni, Pb and Zn) as well as pH were analysed. The element concentrations were measured using inductively coupled plasma optical emission spectrometry (ICP-OES). The materials examined were neutral to ultra-alkaline. Total accumulations (mg kg^−1^) of chemical elements in the soil vary from 130.24 to 14076.67 for Ca, 41.40–926.23 for Na, 0.03–3.34 for Cd, 0.94–103.62 for Cr, 0.94–35.89 for Ni, 3.51–76.47 for Pb and 12.05–279.13 for Zn, whereas quantities of the same elements in the waste samples vary from 171705.13 to 360487.94 for Ca, 517.64–3152.82 for Na, 0.2–9.89 for Cd, 1.16–20.40 for Cr, 1.08–9.79 for Ni, 0.1–146.05 for Pb and 10.26–552.35 for Zn. The vertical distribution of the metals was determined in each soil profile. Despite enrichment of heavy metals in the uppermost horizon on the top of the heap, the results lead to the conclusion that the relation of historical production of soda ash in Jaworzno to current contamination of the local soil environment is insignificant.

## Introduction

Soil is exposed to anthropogenic pollutants, mostly accumulated from ground water and as a result of atmospheric precipitation (>90 %; Stuczyński et al. 2004). Calcium, sodium, chromium, nickel and zinc elements represent macro- and microelements that are essential in the metabolic processes for plants and animals (Kabata-Pendias and Pendias 1999). Contamination of the biosphere by heavy metals (e.g. Cd, Cr, Ni, Pb and Zn) will, if introduced in large amounts, disturb the chemical equilibrium of the natural environment (Kabata-Pendias and Pendias 1999), and may be a danger for living organisms (Senesi et al. 1999; Sivakumar and Subbhuraam 2005; Kachenko and Singh 2006; Sherene 2010; Chudzińska et al. 2014). Ca, Na, Cd, Cr, Ni, Pb and Zn are common components of the Earth’s crust with abundances in upper continental crust rocks’ mass of 3 wt% for Ca, 2.89 wt% for Na, 98 mg kg^−1^ for Cd, 83 mg kg^−1^ for Cr, 44 mg kg^−1^ for Ni, 17 mg kg^−1^ for Pb and 71 mg kg^−1^ for Zn (Taylor and McLennan 2009).

The implementation of the Solvay process in the nineteenth and twentieth centuries was a considerable step towards the ecologization of the production of soda ash (Steinhauser 2008). However, the technology produces copious amounts of solid- and liquid waste and it is still an energy-intensive method (Steinhauser 2008). Large quantities of sodium chloride (NaCl), limestone (CaCO_3_) and ammonia (NH_3_) are consumed as primary components of the ammonia-soda process (Steinhauser 2008). Solid- and liquid effluents (Jadeja and Tewari 2007; Steinhauser 2008), emissions of numerous toxic substances such as heavy metals and organochlorine compounds (Hong et al. 2014), were and are pollutants generated by this industry. The raw materials used, i.e. limestone, coke and brine, are the main source of heavy metals (Integrated Pollution Prevention… 2007).

The fact that scientific data on the impact of the insoluble, solid wastes on the environment are very limited (Steinhauser 2008) encouraged us to investigate the potential influence of solid waste produced by the soda ash plant in Jaworzno on neighbourhood quality. This is the first study of its kind carried out in the environs of the over 100-year-old remains of the soda ash Solvay factory there. The aim of the research was to establish if the historical soda ash production and the naturally reclaimed waste heap have left any recognisable imprint on the local soil. To that end, the heavy metal contents in waste and soil were examined and compared.

## Materials and methods

**Fig. 1:**
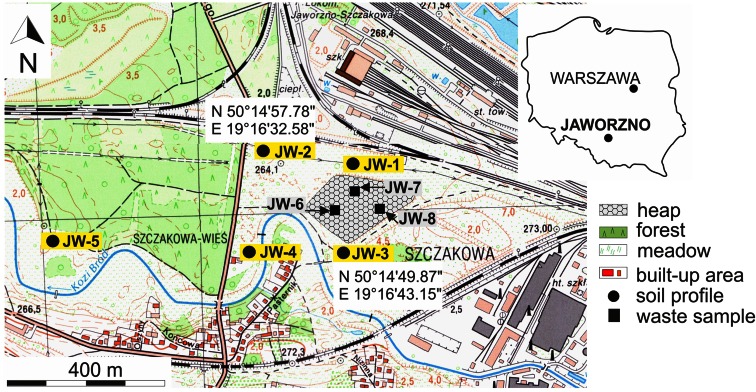
Location of the study area and sampling sites in Jaworzno town

**Fig. 2:**
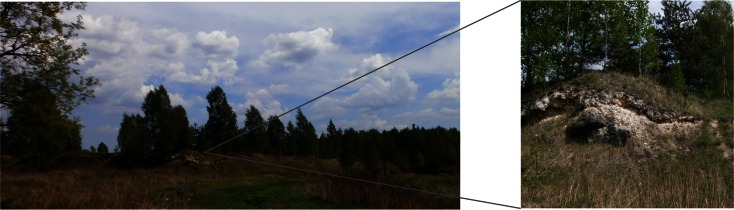
Solvay process waste heap in Jaworzno town

The focus of the research is the anthropogenically transformed district of Jaworzno town in the eastern part of the Upper Silesian industrial region, Southern Poland. Soda ash production by the Solvay method took place there from 1885 to 1909 (Czerwonka 1997; Cohn et al. 2001). The heap, which is a leftover from that period, occupies approximately 3.3 ha and varies in height from 3 to 5 m (Figs. 1 and 2). A soil layer ~10 cm thick covers its top surface, and slag builds up the slopes. Natural reclamation of the site has been ongoing over the last hundred years, and now a variety of plants, including rare- and protected species, inhabit the heap (Tokarska-Guzik et al. 2011). This chemical waste dump is surrounded by meadows, forests, built-up and industrial areas.

Research was conducted on five meadow soil profiles of the podzol developed on fluvio-glacial sediments, and on 18 waste samples collected from three waste locations (Fig. 1). Each soil profile was divided into separate horizons sampled twice, giving 60 soil samples in all. All soil- and waste samples, ~2 kg weight each, were collected with a small spade and stored in plastic sacks.

The samples were oven-dried at 105 °C to constant weight, sieved to 2 mm through a stainless steel sieve and milled into a fine powder. The soil granulometric composition was determined according to Polish Standards (1988). Soil- and waste pH was measured potentiometrically in 1 M KCl solution.

To determine total contents of Ca, Na, Cd, Cr, Ni, Pb and Zn, the samples were digested in a mixture of 6 cm^3^ of concentrated nitric acid and 2 cm^3^ of hydrochloric acid and using a Multiwave 3000 Microwave Digestion (Perkin Elmer) in two steps according to the programme of mineralisation recommended by the equipment provider (power—1400 W, recovery time—5 min, hold—25 min in the first step and 10 min in the second, fan speed—1 in the first step, 3 in the second). After mineralisation, the samples were removed to measuring flasks (10 cm^3^) with 1 % solution of Suprapur nitric acid.

The heavy-metal contents were determined using an inductively coupled plasma optical emission spectrometry (ICP-OES) Optima 7300 Dual View Perkin Elmer atomic emission spectrometer. Each soil analysis was replicated. If the results of the replications differed by more than 5 %, a further analysis of that sample was conducted. The numbers given in Tables 1 and 2 are average values derived from the results obtained for each soil/waste horizon.

**Table 1:** Total metal concentrations (arithmetic means) in soil profiles

Profile number	Depth	Ca	Na	Cd	Cr	Ni	Pb	Zn
	cm	mg kg^−1^	mg kg^−1^	mg kg^−1^	mg kg^−1^	mg kg^−1^	mg kg^−1^	mg kg^−1^
JW-1	0–2	2687.04	143.97	3.34	16.20	14.83	76.47	250.79
	2–7	1892.16	249.43	2.49	8.42	3.25	62.56	217.88
	7–20	817.59	265.43	0.89	3.36	2.37	22.79	84.28
	20–22	510.02	169.17	0.65	2.31	1.50	19.17	64.81
	22–40	1239.13	193.95	0.11	1.09	0.70	3.79	14.55
	40–50	362.29	89.24	0.10	1.09	0.68	2.81	9.29
	50–68	1941.19	125.01	1.51	3.46	2.45	52.68	138.04
	>68	277.12	142.52	0.03	1.08	0.21	1.51	4.84
JW-2	0–1	1605.15	83.72	1.27	5.61	1.70	35.17	120.90
	1–21	561.58	123.99	0.33	10.85	35.20	15.45	51.39
	21–28	808.43	222.38	0.18	3.35	7.29	7.53	24.44
	28–32	3652.98	300.01	1.40	4.13	2.06	37.64	135.69
	32–77	3088.77	926.23	0.88	12.95	10.15	61.61	279.13
	>77	2555.69	168.75	0.10	2.17	1.39	7.56	37.20
JW-3	0–2	5870.07	256.35	1.21	11.80	3.53	36.33	153.87
	2–8	8223.72	382.67	1.09	7.99	2.82	31.63	124.67
	8–18	8826.18	384.54	1.09	14.25	35.89	29.53	120.43
	18–23	8141.92	234.58	1.01	8.33	13.41	27.48	114.22
	23–33	14076.67	409.08	0.67	5.48	4.96	27.52	117.41
	33–37	5961.14	578.36	0.33	1.09	0.21	10.36	41.34
	>37	856.95	155.54	0.07	0.95	0.02	3.51	12.05
JW-4	0–2	735.09	41.40	0.67	103.62	3.06	21.05	120.46
	2–20	735.53	234.41	0.45	89.49	1.45	14.56	93.05
	20–45	871.85	208.60	0.32	89.31	1.31	11.60	81.24
	45–78	414.56	223.90	0.09	8.29	0.18	3.47	17.20
	>78	293.81	720.97	0.06	0.94	0.12	1.33	8.09
JW-5	0–2	968.37	498.24	2.02	6.74	2.41	58.23	196.04
	2–12	760.74	70.79	2.34	7.41	2.90	68.04	223.91
	12–16	463.02	75.91	0.89	2.52	1.21	23.01	85.54
	16–127	130.24	163.51	0.03	1.73	2.27	1.88	7.82
Geometric mean		1342,29	205.73	0.45	5.41	1.86	15.28	60.81
Median		920,11	215.49	0.67	5.55	2.32	21.92	89.30
Relative standard deviation		3291,31	196.51	0.82	26.95	8.85	21.87	76.35
Geom. mean for topsoils in studied topsoil		1712,44	181.49	1.17	12.69	4.54	34.06	133.66
Median for studied topsoils		1286,76	241.92	1.15	9.64	2.98	33.40	122.79
Reference materials								
Median for topsoils South Poland (Pasieczna 2008)		1600		2	6	5	84	200
Median for topsoils Poland (Lis and Pasieczna 1995)		1800		<0.5	4	4	13	35
Geometric mean for Polish podzol and sandy soils (Kabata-Pendias 2001)				0.07	51	7	16	24
Quality standards for soil and ground defined for group B/C (Regulation of the Minister…^2002^)				4/15	150/500	100/300	100/600	300/1000

**Table 2:** Total metal concentrations (arithmetic means) in waste

Profile number	Depth	Ca	Na	Cd	Cr	Ni	Pb	Zn
	cm	mg kg^−1^	mg kg^−1^	mg kg^−1^	mg kg^−1^	mg kg^−1^	mg kg^−1^	mg kg^−1^
JW-6	0–2	171705.13	1263.73	7.48	20.37	9.15	124.57	480.68
	2–15	228673.56	1501.97	9.89	14.56	9.79	146.05	552.35
	15–35	311188.94	1302.58	1.69	4.66	4.42	15.58	126.58
	35–90	278503.81	517.64	1.91	5.31	5.69	17.60	178.85
	90–184	317239.25	731.94	3.74	8.22	6.40	14.01	173.90
	184–207	325043.93	623.23	2.20	6.98	5.20	17.03	154.32
JW-7	0–2	194593.20	1480.00	7.94	20.40	9.55	131.28	436.58
	2–10	272394.29	2057.42	3.72	4.56	3.60	61.20	388.91
	10–23	307449.74	833.38	2.00	5.47	4.99	20.25	160.54
	23–26	345643.43	1941.38	1.07	2.72	2.90	10.17	82.87
	26–46	294949.00	840.87	1.93	4.65	4.17	16.80	130.88
	46–90	290387.23	658.19	2.06	7.54	5.02	13.09	168.21
	90–180	292358.14	858.62	1.44	5.72	3.99	8.55	126.80
	180–230	300556.59	573.93	1.96	6.26	4.38	9.86	177.73
JW-8	0–2	239135.66	2239.37	4.88	15.38	6.04	81.58	248.35
	2–10	277234.18	2755.34	4.97	9.47	5.61	72.03	237.04
	10–30	349348.44	3152.82	0.38	1.66	1.68	2.50	12.89
	10–70	360487.94	3024.32	0.20	1.16	1.08	0.10	10.26
Geometric mean		281626.31	1237.24	2.27	6.24	4.60	18.29	149.59
Median		293653.57	1283.16	2.03	5.99	5.00	16.92	171.05
Relative standard deviation		49598.51	847.73	2.66	5.67	2.35	46.89	149.34

## Results and discussion

The results provide information about the contents of Ca, Na, Cd, Cr, Ni, Pb and Zn and their distribution pattern in the soil profiles and waste samples. The meadow soils (podzols) developed on Pleistocene glacial rocks, mainly sands or clays. The waste material is leftover from the Solvay soda ash process.

The granulometric analysis of the soils shows that they comprise 0.03–10 % gravel, 72.97–99.71 % sand and 0.08–5.7 % silt/clay. According to the classification of texture in soil and mineral materials recommended by the Polish Society of Soil Science (2008), the analysed soils classify as sand.

The pH values measured in the soils vary from 5.94 to 8.68 (median 7.39), whereas the waste material values range from 7.45 to 11.75 (median 8.28). The soils are moderately acidic to strongly alkaline, mostly neutral. The surface layer of the heap is slightly alkaline while the waste samples are strongly alkaline. The results confirm those of Skrzypczak et al. (2009) from the “Biale Morza” ponds of the “Solvay” Krakow Soda Works. According to Kabata-Pendias and Pendias (1999) and Kabata-Pendias (2001), the neutral- and alkaline pH of the soils and waste may affect the immobility of heavy metals, in this case, Cd, Cr, Ni, Pb and Zn.

### Metal contents in soil and waste

Total metal concentrations in the soil and waste are listed in Tables 1 and 2. The soils contain (in mg kg^−1^) 130.24–14076.67 of Ca, 41.40–926.23 of Na, 0.03–3.34 of Cd, 0.95–103.62 of Cr, 0.02–35.89 of Ni, 1.33–76.47 of Pb and 4.84–279.13 of Zn, with geometric means of 1342.29, 205.73, 0.45, 5.41, 1.86, 15.28 and 60.81, respectively. The waste contains (in mg kg^−1^) 171705.13–360487.94 of Ca, 517.64–3152.82 of Na, 0.2–9.89 of Cd, 1.16–20.40 of Cr, 1.08–9.79 of Ni, 0.1–146.05 of Pb and 10.26–552.35 of Zn, with geometric means of 281626.31, 1237.24, 2.27, 6.24, 4.60, 18.29 and 149.59, respectively.

The Ca contents detected in the Jaworzno soils (Table 1) fall into the 700 to 3600 mg kg^−1^ range determined by Zawadzki (1999) for Polish soils. The median value of 1342.29 mg kg^−1^ (Table 1) is lower than that determined for topsoils in Southern Poland (1600 mg kg^−1^; Pasieczna 2008) and for topsoils all over the country (1800 mg kg^−1^; Lis and Pasieczna 1995). The Na contents in the soils are lower than is generally observed in Polish soils (1800–3700 mg kg^−1^; Zawadzki 1999). In the waste samples, Ca contents are much higher as indicated by a calculated median value of 293653.57 mg kg^−1^. Comparison of Ca and Na median values for soil and waste does not demonstrate any obvious increase of either element in soil as a result of mobilisation from the nearby waste.

**Fig. 3:**
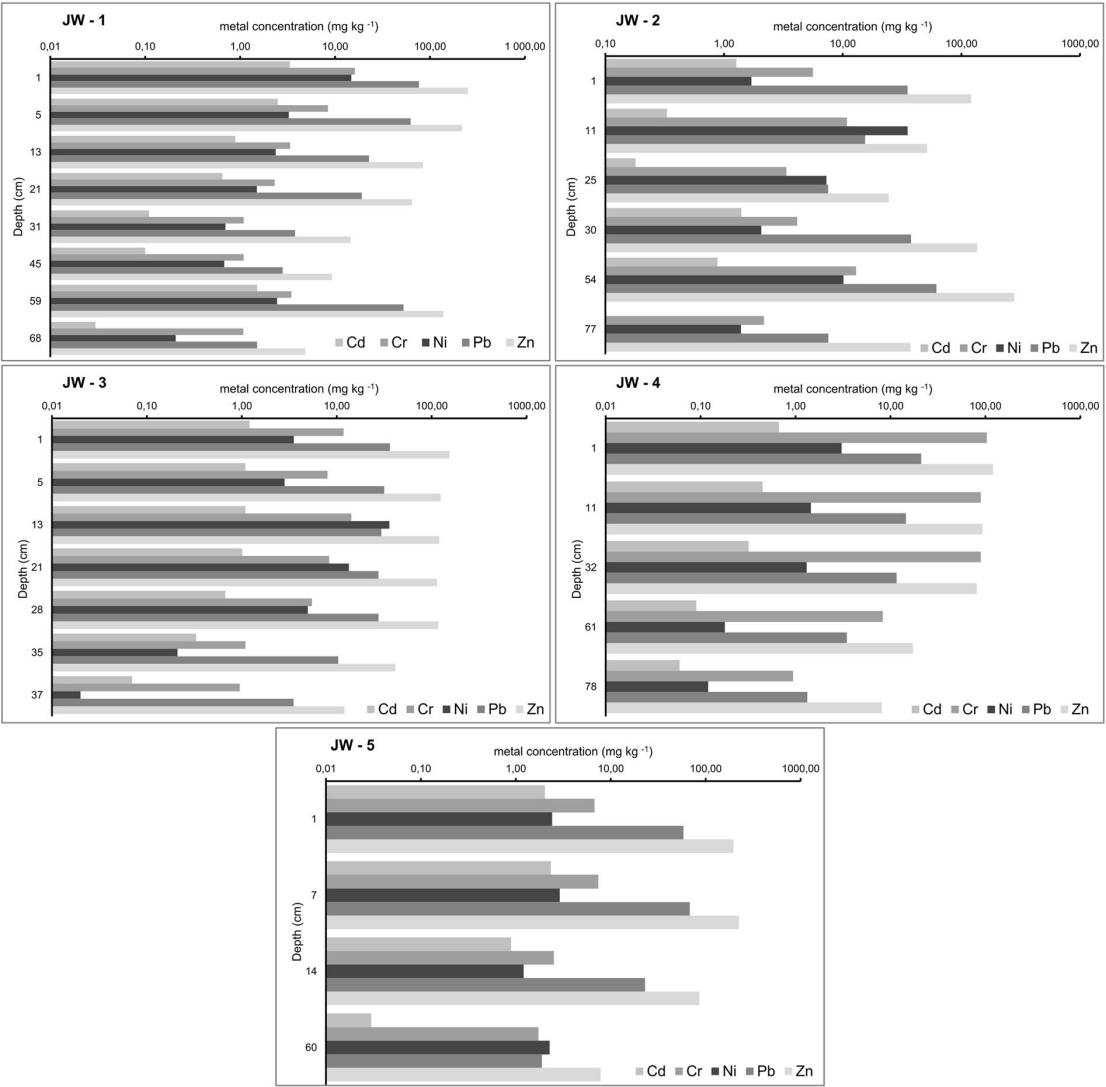
Vertical distribution of heavy metal concentrations (mg kg^−1^) in soil profiles

**Fig. 4:**
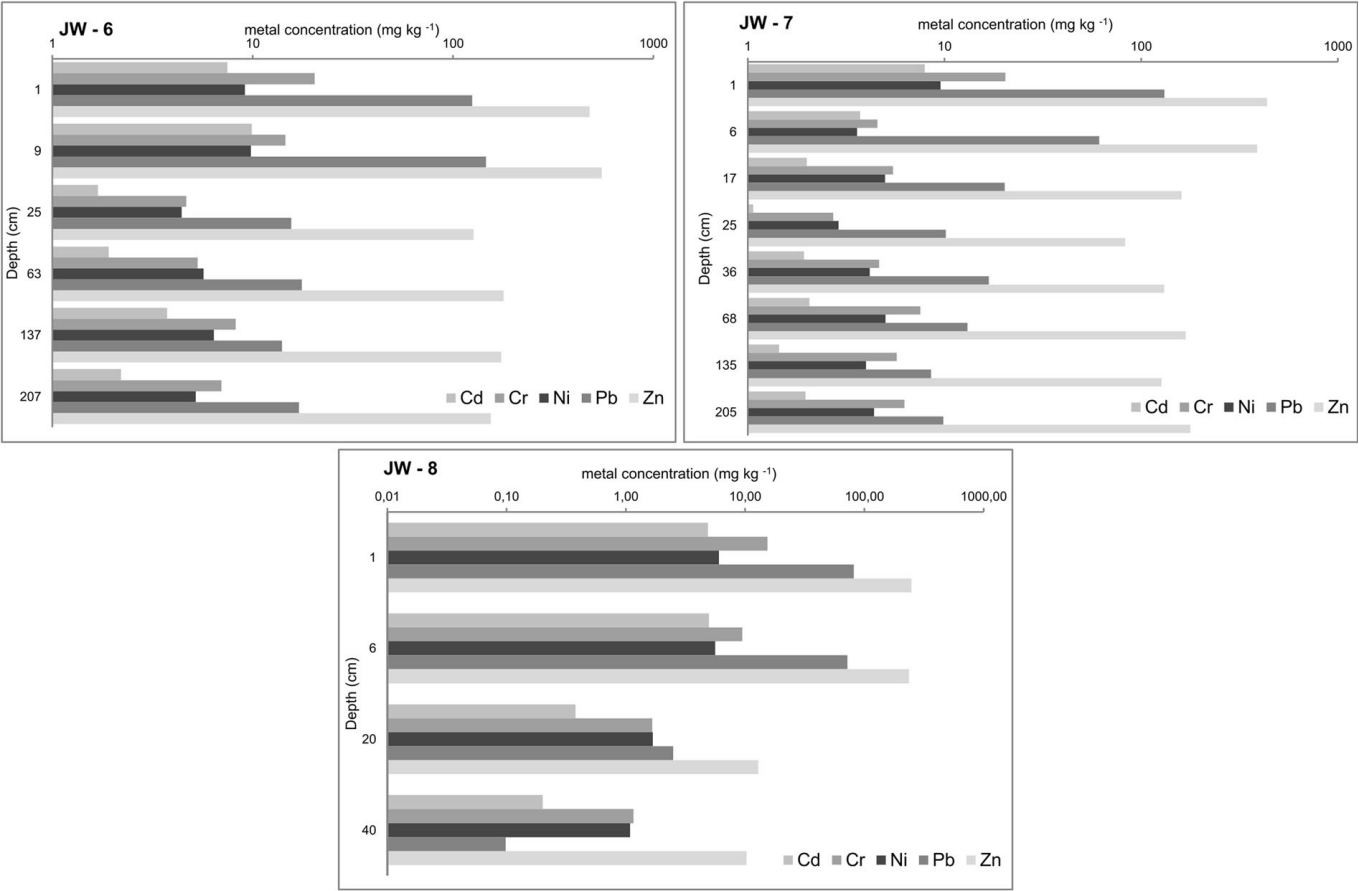
Vertical distribution of heavy metal concentrations (mg kg^−1^) in waste

Generally, metal contents decrease with depth (Figs. 3 and 4). Surface soils, especially that on the top of the waste heap, are enriched in heavy metals (Tables 1 and 2). Metals in the uppermost soil horizons probably originate from coal combustion (Kabata-Pendias and Pendias 1999; Sutkowska et al. 2013), and their elevated levels may result from either adsorption onto soil particle surfaces, co-precipitation with hydroxide or carbonate phases, occlusion in iron/manganese (oxy)hydroxides as coatings on soil particles, binding in lattice positions in aluminosilicates or sorption by organic soil components, reducing solubility and lowering their availability to plants (Tessier and Campbell 1987; Kabata-Pendias and Pendias 1999; Kabata-Pendias 2001; Carrillo‐González et al. 2006). Elevated accumulations of Pb and Zn occurring in the lower part of soil profiles JW-1 and JW-2 at depths of ca 55–60 cm (Fig. 3) are exceptional cases that are possibly due to acid-rain falls in the Upper Silesia in the 1970s and 1980s (Leśniok 2011) causing metal migration into deeper horizons—horizons also enriched in Fe.

The heavy-metal loading determined for the Jaworzno soils meets the quality standards established for soil and ground in Poland (Regulation of the Minister… 2002; Group B—arable lands, forest lands, wastelands; depth of 0.0–0.3 m u.s.l.). With the exception of Cr, almost none of the analysed metal contents exceed the median value determined for topsoils in industrially polluted southern part of Poland (Pasieczna 2008). On the contrary, median values for Cd, Cr, Pb and Zn calculated for the soils analysed here are higher than the medians reported for topsoils all over the country (Lis and Pasieczna 1995).

Soda ash waste profiles (Fig. 4) show decreasing heavy-metal contents with depth (Table 2), identical to that in adjacent soils. The highest levels of the elements measured are present in the thin layer of topsoil. No values exceed the Polish quality standards established for soil and ground allocated to group C (Regulation of the Minister… 2002; industrial areas, mining lands, communication lands; depth of 0.0–0.3 m u.s.l.). The anthropogenic soil overlying soda ash waste is severely contaminated with heavy metals. Median values for Cd, Cr, Pb and Zn contents calculated for this soil are 1.5 to 12 times higher than medians reported for topsoils all over the country (Lis and Pasieczna 1995; Pasieczna 2008).

Contents of some metals in the soils are strongly correlated, e.g. Cd and Pb (0.94), Cd and Zn (0.86) and Pb and Zn (0.96), while other metal pairs are weakly correlated (from −0.15 to 0.16). In the waste, strong positive correlations characterise Cd and Cr (0.89), Cd and Ni (0.91), Cd and Pb (0.97), Cd and Zn (0.94), Cr and Ni (0.90), Cr and Pb (0.88), Cr and Zn (0.82), Ni and Pb (0.84), Ni and Zn (0.86) and Pb and Zn (0.93). In addition, strong negative correlations are found between Ca and the heavy metals, e.g. Ca and Cd (−0.87), Ca and Cr (−0.93), Ca and Ni (−0.87), Ca and Pb (−0.89) and Ca and Zn (−0.89).

The correlations reveal associations between the heavy metals in the waste and the metals in the raw material used in the Solvay plant. Cd, Cr, Ni, Pb and Zn contents correlate strongly among themselves across all of the pairs, suggesting the industrial reworking of metals that occur naturally in the limestone, coke and brine used in the soda ash production. According to a European Commission document on pollution (Integrated Pollution Prevention… 2007), approximately 73 % of the polluting heavy-metals linked to soda ash production is derived from limestone and 21 % from the fuel used for burning the raw materials. Our results are consistent with this proportion when the level of enrichment of metals in the topsoil on the waste heap is compared to their contents in topsoils in the adjacent area where mostly fuel-sourced metals occur (Sutkowska et al. 2013). Furthermore, the strong negative correlations of Ca with Cd, Cr, Ni, Pb and Zn are an indication that the alkalinity of the waste promotes immobilisation of the analysed elements and limits the migration of pollutants. What supports this statement is that the groundwater analysed in the vicinity of the heap (Szulik 2007) meets high standards for satisfactory condition of groundwater (Regulation of the Minister… 2008), and even drinking water (Regulation of the Minister… 2000), in terms of the metal concentrations and pH.

Despite the marked heavy-metal contamination, many plant species, some strictly protected, have spontaneously colonised the surface of the heap. These include species such as *Botrychium lunaria*, *Carlina acaulis*, *Centaurium erythraea subsp. erythraea*, *Centaurium pulchellum*, *Dactylorhiza majalis*, *Epipactis atrorubens*, *Epipactis helleborine*, *Epipactis palustris*, *Gymnadenia conopsea* and *Tofieldia calyculata* (Cohn et al. 2001; Tokarska-Guzik et al. 2011). The behaviour of these metallophytes merits further biogeochemical examination. Currently, investigation of their habitats is ongoing (Rożek 2014).

## Conclusions

This study has shown that the soil environment in the vicinity of an over hundred-year-old heap of soda ash waste is marked by the impact of long-lasting industrial activity. Median values of metal contents for topsoils there are higher than those of Polish soils generally (Lis and Pasieczna 1995). Uppermost soil horizons are weakly enriched in heavy metals. That deeper parts are not so is most probably due to soil alkalinity. The vertical distribution of Cd, Cr, Ni, Pb and Zn contents in the soil profiles indicates an anthropogenic origin for the soil contamination, and the levels of metal content suggest a link to the burning of fossil fuels.

The degree of enrichment of metals in topsoil developed on the waste heap confirms the raw material and fuel used by the Solvay plant as the metal source. The strong alkalinity of the waste and its high Ca content favour retention of metals in the heap, limiting their migration into adjacent soils.

Despite the enrichment of heavy metals in the uppermost horizon on the top of the heap, the results lead us to conclude that the historical production of soda ash has currently an insignificant influence on the local soils. In this regard, the heap in Jaworzno differs from other industrial landfills in the area that contain alkaline wastes (Teper 2009; Chrastný et al. 2012).

Further research to test the bioavailability of the pollutants deposited on the heap is recommended. Such might enable botanists to better understand the habitat of the rare plants spontaneously developed on the soda ash waste heap.
